# Neurogranin and Neuronal Pentraxin Receptor as Synaptic Dysfunction Biomarkers in Alzheimer’s Disease

**DOI:** 10.3390/jcm10194575

**Published:** 2021-10-02

**Authors:** Maciej Dulewicz, Agnieszka Kulczyńska-Przybik, Agnieszka Słowik, Renata Borawska, Barbara Mroczko

**Affiliations:** 1 Department of Neurodegeneration Diagnostics, Medical University of Bialystok, 15-269 Bialystok, Poland; agnieszka.kulczynska-przybik@umb.edu.pl (A.K.-P.); renata.borawska@umb.edu.pl (R.B.); mroczko@umb.edu.pl (B.M.); 2 Department of Neurology, Jagiellonian University, 30-688 Krakow, Poland; slowik@neuro.cm-uj.krakow.pl; 3 Department of Biochemical Diagnostics, Medical University of Bialystok, 15-269 Bialystok, Poland

**Keywords:** neurogranin, neuronal pentraxins receptor, CSF biomarkers, synaptic proteins, Alzheimer’s disease, patients

## Abstract

Synaptic loss and dysfunction are one of the earliest signs of neurodegeneration associated with cognitive decline in Alzheimer’s disease (AD). It seems that by assessing proteins related to synapses, one may reflect their dysfunction and improve the understanding of neurobiological processes in the early stage of the disease. To our best knowledge, this is the first study that analyzes the CSF concentrations of two synaptic proteins together, such as neurogranin (Ng) and neuronal pentraxins receptor (NPTXR) in relation to neurochemical dementia biomarkers in Alzheimer’s disease. Methods: Ng, NPTXR and classical AD biomarkers concentrations were measured in the CSF of patients with AD and non-demented controls (CTRL) using an enzyme-linked immunosorbent assay (ELISA) and Luminex xMAP technology. Results: The CSF level of Ng was significantly higher, whereas the NPTXR was significantly lower in the AD patients than in cognitively healthy controls. As a first, we calculated the NPTXR/Ng ratio as an indicator of synaptic disturbance. The patients with AD presented a significantly decreased NPTXR/Ng ratio. The correlation was observed between both proteins in the AD and the whole study group. Furthermore, the relationship between the Ng level and pTau181 was found in the AD group of patients. Conclusions: The Ng and NPTXR concentrations in CSF are promising synaptic dysfunction biomarkers reflecting pathological changes in AD.

## 1. Introduction

Alzheimer’s disease (AD) is the most common neurodegenerative disease dependent on many neuropathological processes [1,2]. One of the earliest symptoms of Alzheimer’s disease is cognitive impairment, including memory disturbances [3,4]. Memory and learning processes are associated with neuronal communications and hippocampal functions maintained by synapses [3,5]. Impairment of cognitive deficits in AD is associated with neuronal transmission between synapses and neurodegenerative changes [3,6]. The research focused on finding functional pre- and post-synaptic proteins that can contribute to a better understanding of neurobiological mechanisms of AD and improve early diagnosis [3,7]. One of the most important processes involved in memory is long-term potentiation (LTP) and long-term depression (LTD) [8,9]. These two processes are closely related to the increased or decreased intensity of synaptic transmission regulated by synaptic proteins and many other factors [9,10]. Studies on animal models and cell lines have shown how important LTP and LTD are for memory [11,12,13,14]. It is well known that LTP is a neuronal mechanism that underlies memory formation and learning, resulting in an increase in the intensity of synaptic transmission. As shown by studies based on neuronal cell activity registration, one of the factors modulating the LTP mechanism is the Ca2 +/calmodulin (CaM) signaling pathway, which regulates synaptic enhancement through CaMKII, PKC and synaptic proteins activity [11]. Disturbed LTP in the CA1 hippocampus was also observed in an APP/PS1 Mouse Model and other animal models of AD [12,13]. The LTP as a cellular counterpart to memory can be modulated by several different synaptic pathways, including those associated with Ca2+/CaM, as well as neurogranin and neuronal pentraxins [10,14,15]. Therefore, it seems particularly important to study synaptic proteins as biomarkers of AD disease. Nevertheless, these processes are still not yet fully understood and explained in neurodegenerative disorders.

The literature data indicate that impaired synaptic transmission may be caused by various forms of amyloid β (Aβ), one of AD’s most important causative factors [16,17,18,19]. The Aβ1-42 and small oligomeric forms (Aβo) disrupt LTP, probably by interacting with the N-methyl-D-aspartate receptor (NMDAR), leading to synaptic loss and neuronal death [12,20,21,22]. On the other hand, tau and their small forms may interfere with an α-amino-3-hydroxy-5-methyl-4-isoxazolepropionate receptor (AMPAR) and NMDAR, leading to impaired glutamatergic transmission in excitatory neurons in crucial brain regions, such as the hippocampus [10,23].

In general, both receptors AMPA and NMDA play an essential role in LTP by opening Na+ and Ca2+ channels in response to glutamate [24,25]. In Alzheimer’s disease, there is a far more progressive glutamatergic dysfunction associated with both receptors [10,16]. The AMPA, the principal ionotropic receptor, works faster and shorter, especially when there is a small amount of glutamate and excitability [24]. The NMDAR acts slower and longer, which depends on sufficiently strong depolarization and synaptic release of glutamate [25,26]. The cooperation between AMPARs and NMDARs is required to respond to post-synaptic membrane depolarization and ions diffusion [24]. Increased intracellular Ca2+ concentration in post-synaptic neurons provides numerous biochemical processes necessary for LTP induction [25,27]. It has been suggested that synaptic proteins may modulate LTP through interaction via the calcium (Ca2+)/calmodulin (CaM) pathway and NMDARs [10]. On the other hand, AMPAR’s function may be regulated, e.g., by binding proteins [28]. The imbalance of homeostatic mechanisms between excitatory and inhibitory synapses plays a critical role in contributing to the cognitive decline in AD patients [16,29].

Considering the mentioned facts seems crucial to study proteins reflecting synaptic dysfunctions in AD. Over the last few years, promising results have emerged regarding biomarkers of synaptic dysfunction, including pre-synaptic proteins (Synaptosomal-Associated Protein (SNAP-25), synaptotagmin-1, or Growth Associated Protein 43 (GAP-43)) and post-synaptic molecules (Neurogranin (Ng)) [30,31,32,33,34,35], as well as indicators of synaptic functioning (Neuropentraxins family proteins (NPTX)) or neurotransmission (Synaptic vesicle glycoprotein 2A (SV2A), Glutamate Ionotropic Receptor AMPA Type Subunit 4 (GRIA4)) [36,37,38,39]. A study conducted by Leo et al. revealed the clinical usefulness of few synaptic proteins in periclinal stages of AD [38]. It is difficult to clearly identify which of the above synaptic proteins will be accurate and specific for AD pathology due to still ongoing research. However, an increasing interest in CSF synaptic biomarkers has been observed due to the early manifestation of synaptic loss in cognitive decline pathology [40]. The changes in the concentrations of these proteins may be an indicator of early synaptic dysfunction [3,7]. Therefore, we examined the concentrations of the following two proteins associated with synaptic plasticity and glutamatergic receptors: neurogranin (Ng) and neuronal pentraxin receptor (NPTXR). Neurogranin is a post-synaptic protein mainly expressed in pyramidal cells of the hippocampus, cortex and highly concentrated in dendritic spines [41,42,43]. Many studies suggest that Ng is involved in regeneration synapses, synaptic plasticity and LTP induction by Ca2+ and CaM signaling pathways [10,15,44]. The function of neurogranin is closely related to NMDAR [10,41]. Zhong and Gerges suggested that Ng regulates metaplasticity by regulating or targeting CaM localization in dendritic spines, which translates into LTP and LTD modulation [44]. The loss of dendritic spines and synapses may be closely related to the increased levels of Ng in CSF [10]. The increased concentration of Ng was observed in CSF patients with mild cognitive impairment (MCI) and AD [34,45,46,47,48]. Notably, other authors confirm the relationship between CSF elevated Ng levels and atrophy of brain structures, such as the hippocampus, lateral ventricles and loss of the whole brain volume in MCI and AD patients [45,48,49]. A summary of the general upward trend of Ng in CSF patients with AD and MCI was presented in our meta-analysis [50]. That, in turn, maybe one of the earliest molecular mechanisms of synaptic neurodegeneration.

The NPTXR is a unique transmembrane protein from the neuronal pentraxins family [51,52]. The highest expression and involvement in neuronal processes of NPTXR was observed in the hippocampus and cerebral cortex [29,51]. It has been suggested that NPTXR organized synaptic maturity, plasticity and clustering to AMPAR, influencing synaptic transmission [14,29,53]. Additionally, NPTXR may recruit AMPAR into glutamatergic synapses, crucial for LTP [14,53,54]. In the literature, only a few articles are available concerning the NPTXR levels in the CSF of AD patients [36,55,56]. Begcevic et al. also observed reduced NPTXR levels in the CSF of AD patients [55]. The authors assessed 30 brain-specific proteins using mass spectrometry, and in the second step, they confirmed the results using an ELISA. The researchers reported that NPTXR reflects the AD severity and is the most promising biomarker [55]. These findings were supported by a study conducted by Lim et al., where the decreased levels of NPTXR in AD patients were noted [36]. Moreover, the authors revealed that the levels of NPTXR changed with the dementia severity and progression [36]. In line with that are other findings, which demonstrated the relationship of NPTXR with AB load in the PET study [56].

Both proteins are crucial factors regulating the physiological processes of memory and other cognitive functions. However, their role in cognitive decline and the development of AD is not fully understood. Therefore, in this study we investigate Ng and NPTXR levels in the cerebrospinal fluid of AD patients and analyze their relationship with classical AD biomarkers. It seems that deeper knowing of synaptic pathology allows for a better understanding of neurobiological mechanisms in AD and may improve early diagnosis of the disease.

## 2. Results

### 2.1. The CSF Concentrations of Ng and NPTXR as Synaptic Biomarkers

**Figure 1:**
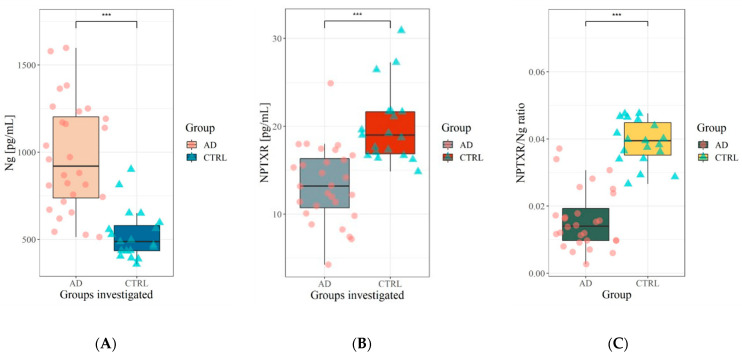
(**A**) Cerebrospinal fluid level of neurogranin in AD and CTRL group; (**B**) Cerebrospinal fluid concentration of neuronal pentraxin receptor in AD and CTRL group; (**C**) NPTXR/Ng ratio in AD and CTRL group. Legend—Level of statistically significant *** *p* < 0.001, Ng—neurogranin, NPTXR—neuronal pentraxin receptor, NPTXR/Ng ratio—neuronal pentraxin receptor to neurogranin ratio, AD—Alzheimer’s disease, CTRL—control, CSF—Cerebrospinal fluid.

The biochemical and demographic characteristics of study participants were presented in Table 1 and Table 2, respectively. The mean age of the AD patients was somewhat higher than the controls but did not differ statistically. Based on the MMSE score, biochemical analyses and clinical picture, we chose patients with not very advanced AD because we aimed to check if the concentrations of selected synaptic proteins may reflect the early synaptic pathology and there is a relationship with amyloid and tau biomarkers in the early phase of full-blown disease. The concentrations of Ng and NPTXR in the cerebrospinal fluid are presented in Table 2. Based on the U-Mann–Whitney test, the significant differences between the tested group were observed for CSF levels of Tau (*p* < 0.001), pTau181 (*p* < 0.001), Aβ42/40 ratio (*p* < 0.001), Aβ42 (*p* < 0.001), Ng (*p* < 0.001) and NPTXR (*p* < 0.001). The Ng levels in CSF differed significantly between the patients with AD and the controls (Table 2, Figure 1). A similar pattern was observed for the CSF levels of NPTXR protein. However, the concentrations of NPTXR were significantly lower in AD than in the controls, and Ng were higher. We calculated the NPTXR/Ng ratio. The AD patients presented a statistically significant decreased NPTXR/Ng ratio as compared with the controls.

### 2.2. Associations between CSF Levels of Ng, NPTXR and Neurochemical Biomarkers (Aβ42/40 Ratio, Tau, pTau181)

**Figure 2:**
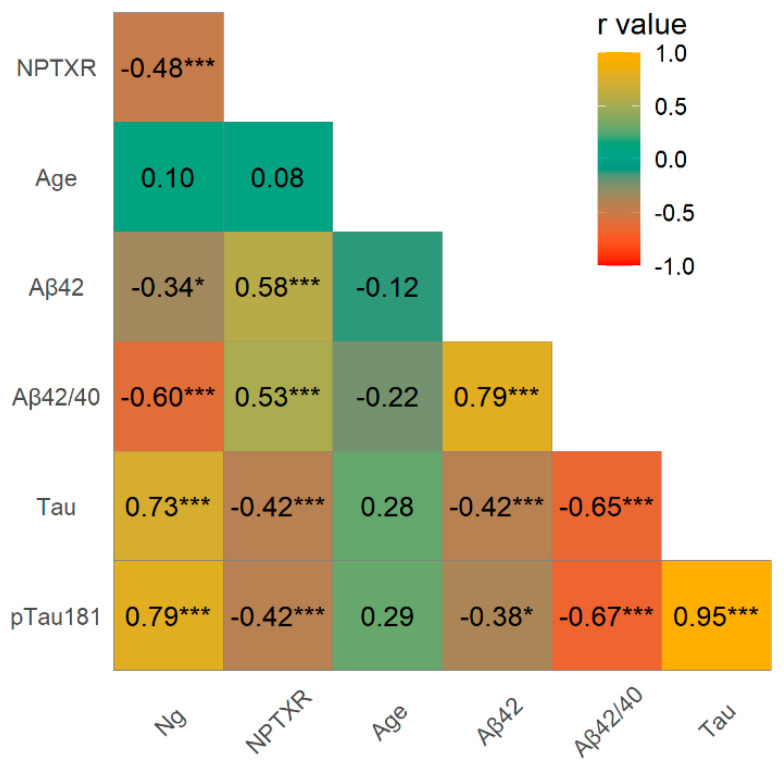
Spearman’s correlations between neurochemical biomarkers and tested proteins in the whole study group. Legend—Level of statistically significant *** *p* < 0.001, * *p* < 0.05, Ng—neurogranin, NPTXR—neuronal pentraxin receptor, Aβ42—amyloid Beta 1-42, Aβ42/40—amyloid Beta 1-42 to 1-40 ratio, AD—Alzheimer’s disease, CTRL—control, CSF—Cerebrospinal fluid.

The associations between levels of Ng, NPTXR and neurochemical biomarkers of AD were performed using the Spearman rank correlation test (Figure 2). Significant positive correlations were observed in the whole study group between CSF Ng and Tau (rho = 0.73, *p* < 0.001), and pTau181 (rho = 0.79, *p* < 0.001), and negative with NPTXR (rho = −0.48, *p* < 0.001), the Aβ42/40 ratio (rho = −0.60, *p* < 0.001), Aβ42 (rho = −0.34, *p* < 0.05) and MMSE (rho = −0.56, *p* < 0.001). A positive correlation was observed between NPTXR and the Aβ42/40 ratio (rho = 0.53, *p* < 0.001), Aβ42 (rho = 0.58, *p* < 0.001), and a negative association between NPTXR and Tau (rho = −0.42, *p* < 0.001), as well as pTau181 (rho = −0.42, *p* < 0.001).

**Figure 3:**
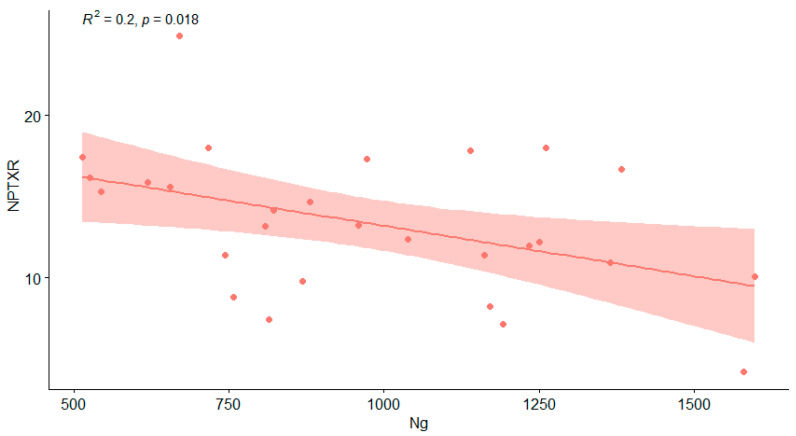
Correlation between CSF Ng and NPTXR levels in the AD group (showed with red dots represents results of each AD patients and line of best fit with 95% CI). Legend: NPTXR—neuronal pentraxin receptor, Ng—neurogranin, AD—Alzheimer’s disease.

In the AD group, the CSF levels of Ng significantly correlated with NPTXR (rho= −0.40, *p* = 0.038) and pTau181 (rho = 0.384, *p* = 0.044) (Table 3, Figure 3).

## 3. Discussion

Synaptic dysfunctions and loss are among the earliest signs of dementia that are closely related to cognitive symptoms underlying the neurobiological processes in AD [3,7,13]. Therefore, it seems important to study the proteins reflecting synaptic dysfunction as indicators of disease progression and developing cognitive disorders. To the best of our knowledge, this is the first study that analyzes the CSF concentrations of two synaptic proteins, such as neurogranin (Ng) and neuronal pentraxin receptor (NPTXR), in relation to neurochemical dementia biomarkers (NDD). Neurogranin and neuronal pentraxin receptors seem to be novel, promising biomarkers that may reflect pathological changes of synaptic disturbance in patients with Alzheimer’s disease [36,45,55].

In agreement with other research, our study confirmed significantly higher concentrations of Ng in the AD group compared with cognitively healthy controls [34,45,46,57,58]. Moreover, our extensive meta-analysis supports the general trend of elevated concentrations of Ng in the CSF of AD patients [50]. It is important to note that high levels of Ng were observed not only in dementia subjects (with AD and MCI), but also in patients with Creutzfeldt–Jakob disease (CJD) [59]. The elevated level of Ng in AD patients may be an indicator of synaptic and dendritic degeneration [60]. Abnormalities of synaptic and dendritic transmission are presented as one of the earliest signs of neurodegeneration and cognitive impairment [21,61,62]. It was reported that increased Ng levels correlated with AD progression, which may indicate its importance as a predictor of developing synaptic pathology [45]. Synaptic disruption is probably due to the pathological effects of short forms of Aβ oligomers by binding and inducing the internalization of NMDAR, which affects the NMDA signaling pathways [10,21,63]. Due to several possibilities of pathological impact, the amyloid molecular signaling and consequences for LTP have yet to be elucidated [21,64]. It is suggested that soluble Aβo induces a loss of glutamatergic synapses and LTP, which reduces the dendritic spines [64,65]. Glutamatergic transmission is one of the first to be disrupted in AD pathology [16,22]. Probably, NMDA receptors are the common denominator of neurogranin and early amyloidosis in glutamatergic neurons [44,66]. An elevated level of Ng appears to be associated not only with synaptic but also with dendritic degeneration [42]. The in situ hybridization study has shown that the Ng mRNA selective translocation to dendrites is impaired in the cortex of AD patients [67]. Probably, Ng was released during the loss of synapses and dendrites.

Despite the fact that neuroimaging studies have shown the relationship of the Ng level with future rate hippocampal atrophy and amyloid load in preclinical AD subjects and AD patients [45,46,57], our study did not reveal any correlation between the levels of Ng and amyloid-beta 1-42 in the AD patients. Similarly, other researchers also did not find significant correlations between Aβ and Ng in the CSF of AD patients [46,60,68,69]. However, experimental models supported the correlation between Ng, the loss of synaptic connections and amyloidosis [70]. Cortical thickness and elevated Ng levels were associated with observable Aβ pathology in the early stages of AD [48,71]. In addition, the co-occurrence of cortical and hippocampal atrophy has also been confirmed in animal models [72,73]. Perhaps circulating amyloid in the CSF and synaptic space forms complexes with other proteins or synaptic receptors, making it impossible to detect using commonly available methods. Supporting this hypothesis is the fact that Aβ not only aggregates but also interacts with NMDAR receptors by binding and disrupting glutamatergic transmission, resulting in neuronal death [10,74]. Likewise, we did not observe a significant correlation between Ng levels and MMSE in the patients with dementia. The findings of other researchers concerning the correlation between Ng and MMSE are also inconclusive [45,46,75]. In the AD group, we observed a significant association between increased Ng and pTau181, which agrees with other investigations [34,45,46,58]. A positive correlation with pTau181 indicates a process of neurodegeneration and microtubular dysfunction, and neuronal death. Some research suggests that soluble Tau may colocalize with synaptic markers into synapses in AD pathology [76,77]. In addition, the pathological role of Tau may be related to the trafficking of neurotransmitters in post-synaptic receptors localized at dendritic spines [78,79]. The correlation with tau may also be related to axonal degeneration and early microtubule breakdown and release at synapses.

In our research, Ng was negatively correlated with NPTXR in the AD patients and the whole study group. We can speculate on the common link between Ng and NPTXR in synaptic pathology in AD. Several arguments and physiological processes seem to indicate a close interaction between these proteins. Both proteins NPTXR and Ng are involved in the LTP processes of glutamatergic synapses [10,29,41]. The AMPARs play a primary role in excitatory synaptic transmission in the hippocampus. NPTXR interacts most strongly with AMPAR channels, but it is not excluded from interacting with inhibitory neurons [29,53,80]. Studies on neuronal cultures show that NPTXR knockdown decreased excitatory synapse organization [53]. Additionally, studies in NPTXR-/- and NPTXR2-/- deletion mice showed significant synaptic impairment due to GluA4 deficiency [29]. This indicates an essential role in GluA4 recruitment for AMPARs and the selective regulation of neuronal networks in the hippocampus [29].

On the other hand, an imbalance between arousal and the inhibition ratio impairs the cognitive and intellectual abilities in people with AD [16]. We observed decreased NPTXR levels in the CSF of AD patients, which may be indirectly related to impaired synaptic transmission and in particular, glutamatergic signaling. Other researchers have shown that NPTXR levels in the CSF changed with disease progression, starting with mild cognitive impairment (MCI) [36,55]. Neuroimaging studies by Lim et al. showed significantly lower levels of NPTXR in Aβ+ (positive) patients than Aβ- (negative) [56]. These studies further support the hypothesis that, similarly to neurogranin, NPTXR may be associated with the Aβ-induced impairment of synaptic transmission.

The association between Ng and NPTXR might be related to the dysfunction of glutamatergic synapses. The combination of two analytes gives statistically significant differences between AD and CTRL. As a ratio, the CSF levels of NPTXR and Ng might be a more specific reflection of synaptic degeneration than the individual analytes separately. The assays to measure AD CSF biomarkers characterize limitations, such as between laboratory and lot-to-lot variation. Therefore, the use of ratios seems to be better for the accurate classification of patients than individual novel biomarkers. Taken together, both proteins are more reliable in reflecting pathological processes inside the synapses. These proteins are also responsible for synaptic transmission in glutamatergic neurons, which is essential in neurodegenerative diseases. As a ratio, the CSF levels of NPTXR and Ng might be a more solid reflection of synaptic dysfunction or integrity than the single measurement of concentration. We were more concerned with the relevance in biomarker studies that would reflect the biological relationship in the context of Alzheimer’s disease. Of course, our observations are a proposition and a challenge for further research. Moreover, our results should be confirmed by other researchers from other centers on larger groups of patients. Moreover, further, more detailed studies on synaptic transmission in AD and MCI should be conducted. It is suggested that both Ng and NPTXR and the proposed NPTXR/Ng ratio may prove to be useful synaptic biomarkers.

## 4. Materials and Methods

### 4.1. Study Population and Diagnostic Criteria

The study population involved *n* = 47 (*n* = 33 women, *n* = 14 men, 70 median years) subjects from the Department of Neurology, Jagiellonian University Hospital, Krakow, Poland, and included 28 AD patients and 19 non-demented controls. In the clinical diagnosis of the study group, standard medical examination, magnetic resonance imaging or computed tomography of the brain, a physical and neurological examination, laboratory screening tests and a comprehensive neurocognitive evaluation were used. The AD diagnosis was based on the recommendations from the National Institute on Aging and Alzheimer’s Association (NIA-AA) criteria [81]. Neuroimaging and neuropsychological examinations were combined with neurochemical findings for the most accurate clinical diagnosis of AD (levels of Aβ1–42, Tau and pTau181, and values of the Aβ1–42/Aβ1–40 ratio). The study was conducted in the Department of Neurodegeneration Diagnostics at the Medical University of Bialystok, according to the guidelines of the Declaration of Helsinki, and was approved by the Ethics Committee of Medical University of Bialystok at 29 November 2018 (R-I-002/459/2018).

Patients with a suspected cerebrovascular disorder, increased albumin quotient (QAlb) indicating blood–CSF barrier dysfunction or alternations in CT/MRI images were excluded from the study. Information about the past medical history of patients was also verified. The biochemical characteristics of study participants based on the concentrations of classical biomarkers for AD and CSF parameters are presented in Table 1. The MMSE score was used to assess dementia severity. The Erlangen Score algorithm for the interpretation of CSF biomarkers was used [82].

The control group consisted of people who did not have subjective memory disorders that did not fulfill the MCI criteria or recurrent headaches. A careful examination of subjects in the control group, with detailed analyses of the CSF, allowed for excluding the symptoms’ organic background. No one in the control group showed any significant alternations in the established biomarkers for AD (levels of Aβ1–42, Tau and pTau181). These findings were confirmed by an Erlangen Score of 0 points in all 19 subjects of this group.

### 4.2. Biochemical Evaluation

After collection, CSF samples were centrifuged, aliquoted and frozen at −80 °C in polypropylene tubes until analysis. The concentrations of tested proteins (Ng, NPTXR, Aβ1–42, Aβ1–40, Tau and pTau181) in CSF were measured in the Department of Neurodegeneration Diagnostics, Medical University of Bialystok, Poland. The quantitative assessment of neurochemical dementia diagnostics (NDD) biomarkers in CSF was performed using IBL kits (Hamburg, Germany) for Aβ42, Aβ40 and Fujirebio kits (Gent, Belgium) for t-tau and pTau181 proteins. The concentrations of NPTXR were assessed with a commercially available RayBioHuman NPTXR ELISA kit (ELH-NPTXR; Ray Biotech, Norcross, GA, USA). The CSF samples were diluted 25-fold in PBS and tested in duplicates. Absorbance was read at 450 nm. The Ng concentrations were assessed using a commercially available quantitative bead-based immunoassay (MILLIPLEX MAP Human Neuroscience Magnetic Bead Panel 2, HNS2MAG-95K, Merck KGaA, Darmstadt, Germany). The assay was performed in agreement with the manufacturer’s instructions, and samples were diluted at 1:10. Washing steps were conducted using Biotek 405LS. For readout, the 96-well plates and a Luminex® 100/200™ analyzer (Luminex Corporation, Austin, TX, USA) were used. Standards and samples were run in duplicates with a coefficient of variance (CV) < 20%.

### 4.3. Statistical Analysis

Statistical analysis and visualization were performed by nonparametric tests and analysis using the *PMCMRplus* and *ggraph2* packages in the free statistical software RStudio: Integrated Development for R. RStudio (Version 1.2.5019), PBC, Boston, MA, USA. The data from the quantitative CSF biomarker did not fit a normal distribution. The concentrations of tested variables in investigated groups were carried out by using a U Mann–Whitney test. The results are presented as medians and interquartile ranges in tables. Statistical significance was set at *p* < 0.05. We analyzed correlations between Ng, NPTXR and the core AD biomarkers via the Spearman rank correlation non-parametric test.

## 5. Conclusions

Ng and NPTXR appear to be promising biomarkers of synaptic degeneration. Our results confirm statistically significant differences between both proteins in the AD patients compared to the controls. According to our best knowledge, this is the first study that compares Ng and NPTXR in CSF with classical AD biomarkers. Considering that Ng positively correlated with pTau181, this protein seems to be a more reliable biomarker of neurodegenerative changes strictly related to synaptic damage. This association may reflect an already advanced process of a loss of synapses and dendritic spines in fundamental brain structures. We concluded that a decrease in the NPTXR/Ng ratio would correspond to the atrophy of synapses and disrupted synaptic transmission. Our results suggest that Ng and NPTXR taken together can be used as additional parameters to assess synaptic dysfunction in the clinical diagnosis of AD patients. We realize that research should be continued on a larger group of patients and confirmed by other researchers. Furthermore, we hope that the proposed analyses may be an essential step in developing diagnostics for synaptic dysfunction.

## Figures and Tables

**Table 1:** Demographic data and characteristics of the study groups.

	Median (Interquartile Range)	
	AD *n* = 28	CTRL *n* = 19
Age (mean in years)	75.5 (65.5–80.5)	67 (64–73)
Gender (Female/Male)	21/7	12/7
MMSE score (range 0–30 p.)	22 (18.8–23)	28.5 (27–30)

Note: AD—Alzheimer’s disease, CTRL—control, MMSE – Mini-Mental State Examination.

**Table 2:** The concentrations of tested proteins in the study groups.

Tested Variables in CSF	Median (Range of Interquartile)		*p* (U-Mann–Whitney)
	AD	CTRL	
Aβ42/40 ratio	0.032 (0.03–0.04)	0.066 (0.06–0.08)	<0.001
Aβ42	513 (460–655)	926 (815–1004)	<0.001
Tau (pg/mL)	676 (591–1058)	222 (191–273)	<0.001
pTau181 (pg/mL)	86.7 (73.2–122)	37.5 (34–42.9)	<0.001
Ng (ng/mL)	920 (737–1202)	487 (435–580)	<0.001
NPTXR (pg/mL)	13.2 (10.8–16.3)	19 (16.9–21.6)	<0.001
NPTXR/Ng ratio	0.014 (0.009–0.019)	0.395(0.039–0.044)	<0.001

Note: Ng—neurogranin, NPTXR—neuronal pentraxin receptor, Aβ42—amyloid Beta 1-42, Aβ42/40—amyloid Beta 1-42 to 1-40 ratio, AD—Alzheimer’s disease, CTRL—control, CSF—Cerebrospinal fluid.

**Table 3:** Spearman’s correlations between CSF tested proteins and neurochemical biomarkers in the AD patients. Legend—Level of statistically significant *** *p* < 0.001, ** *p* < 0.01, * *p* < 0.05, Ng—neurogranin, NPTXR—neuronal pentraxin receptor, Aβ42—amyloid Beta 1-42, Aβ42/40—amyloid Beta 1-42 to 1-40 ratio.

			Spearman’s Rho	*p*	
Ng	-	NPTXR	−0.40	*	0.038
Ng	-	pTau181	0.38	*	0.044
Aβ42	-	Aβ42/40	0.52	**	0.004
Tau	-	pTau181	0.88	***	<0.001

## Data Availability

The data presented in this study are available on request from the corresponding author.

## Abbreviations

AD	Alzheimer’s Disease
AMPAR	α-amino-3-hydroxy-5-methyl-4-isoxazolepropionate receptor
Aβ	amyloid β
Aβo	amyloid β oligomers
CaM	calmodulin
CJD	Creutzfeldt–Jakob disease
CSF	Cerebrospinal Fluid
CT	computer tomography
CTRL	controls
ELISA	enzyme-linked immunosorbent assay
GAP-43	Growth Associated Protein 43
GluA4	glutamate ionotropic receptor AMPA type subunit 4
LTD	long-term depression
LTP	long-term potentiation
MCI	Mild cognitive impairment
MRI	magnetic resonance image
NDD	neurochemical dementia biomarkers
Ng	Neurogranin
NMDAR	N-methyl-D-aspartate receptor
NPTXR	Neuronal pentraxin receptor
PET	Positron Emission Tomography
pTau181	phosphorylation Tau protein (Threonine 181)
SNAP-25	Synaptosomal-Associated Protein 25
